# When will sub-Saharan Africa adopt HIV treatment for all?

**DOI:** 10.4102/sajhivmed.v17i1.459

**Published:** 2016-08-31

**Authors:** Somya Gupta, Reuben Granich

**Affiliations:** 1 International Association of Providers of AIDS Care, New Delhi, India; 2 International Association of Providers of AIDS Care, Washington DC, United States

## Abstract

**Background:**

The World Health Organization (WHO) HIV treatment guidelines have been used by various countries to revise their national guidelines. Our study discusses the national policy response to the HIV epidemic in sub-Saharan Africa and quantifies delays in adopting the WHO guidelines published in 2009, 2013 and 2015.

**Methods:**

From the Internet, health authorities and experts, and community members, we collected 59 published HIV guidelines from 33 countries in the sub-Saharan African region, and abstracted dates of publication and antiretroviral therapy (ART) eligibility criteria. For these 33 countries, representing 97% regional HIV burden in 2015, the number of months taken to adopt the WHO 2009, 2013 and/or 2015 guidelines were calculated to determine the average delay in months needed to publish revised national guidelines.

**Findings:**

Of the 33 countries, 3 (6% regional burden) are recommending ART according to the WHO 2015 guidelines (irrespective of CD4 count); 19 (65% regional burden) are recommending ART according to the WHO 2013 guidelines (CD4 count ≤ 500 cells/mm^3^); and 11 (26% regional burden) according to the WHO 2009 guidelines (CD4 count ≤ 350 cells/mm^3^). The average time lag to WHO 2009 guidelines adoption in 33 countries was 24 (range 3–56) months. The 22 that have adopted the WHO 2013 guidelines took an average of 10 (range 0–36) months, whilst the three countries that adopted the WHO 2015 guidelines took an average of 8 (range 7–9) months.

**Conclusion:**

There is an urgent need to shorten the time lag in adopting and implementing the new WHO guidelines recommending ‘treatment for all’ to achieve the 90-90-90 targets.

## Introduction

The rapid expansion of antiretroviral therapy (ART) has been a cornerstone in transforming the acquired immunodeficiency syndrome (AIDS) response globally. In March 2015, the ’15 by 15’ target set out in the 2011 United Nations Political Declaration on HIV and AIDS was achieved ahead of the deadline.^1^ The greatest gains in access to ART occurred in sub-Saharan Africa, a region with 25.5 million people living with HIV.^2^ However, treatment coverage remains relatively low. In 2015, only 12.1m (47%) people living with HIV in the region were accessing ART.^2^ There were 1.4m (range 1.1m–1.6m) new HIV infections and 800 000 (range 640 000–990 000) AIDS-related deaths.^2^ Barring unforeseen events, the AIDS epidemic will continue to outrun the response and these figures are likely to rise.^3^ To end the AIDS epidemic by 2030, the Joint United Nations Programme on HIV/AIDS (UNAIDS) has established the 90-90-90 targets for 2020 (i.e. 90% of people living with HIV to know their status; 90% of people diagnosed with HIV to be accessing treatment; and 90% of those on ART achieving viral suppression) that will prevent an estimated 21m AIDS-related deaths and 28m new HIV infections worldwide by 2030.^3^ Shortly after these targets were established, the U.S. President’s Emergency Plan for AIDS Relief (PEPFAR) adopted them as part of the pivot to PEPFAR 3.0.^4^ Achieving these targets would require countries to align their national guidelines and programmes with the latest scientific evidence that demonstrates the benefits of immediate ART in reducing the risk of HIV-related morbidity, mortality, transmission and costs.^5,6,7,8,9,10,11,12,13,14^

Translating scientific discovery into policy and then service delivery generally takes years. However, delays translate into missed public health opportunities to prevent illness, death and transmission and deny millions of people timely access to life-saving treatment. Since 2003, the World Health Organization (WHO) has updated its treatment guidelines to reflect expert consensus on when to initiate HIV treatment. Whilst the WHO 2003 guidelines recommended ART at CD4 count ≤ 200 cells/mm^3^ for adults and adolescents living with HIV,^15^ the 2006 guidelines additionally recommended considering ART at CD4 count ≤ 350 cells/mm^3^.^16^ In 2009 and 2013, WHO guidelines updated the ART eligibility criteria to CD4 count ≤ 350 cells/mm^3^ and CD4 count ≤ 500 cells/mm^3^, respectively.^17,18^ Recently, based on results from the HPTN 052,^5^ the INSIGHT-START^6^ and the TEMPRANO trial,^7^ the WHO issued an early release to its guidelines in September 2015 to recommend immediate ART at all CD4 counts.^19^

Most of the countries in sub-Saharan Africa use the internationally accepted WHO guidelines as a reference to revise their national guidelines. Our study looks at the national ART policy response to the HIV epidemic in sub-Saharan African countries, and quantifies delays in national level adoption of the WHO ART guidelines published in 2009, 2013 and 2015.

## Methods

From the International Association of Providers of AIDS Care’s (IAPAC) National Policy Database,^20^ we collected the latest national treatment guidelines for adults and adolescents for 48 countries in sub-Saharan Africa. This database (www.hivpolicywatch.org) is current as of July 2016 and has been constructed over a 5-year period using quarterly Internet searches and direct submissions from Ministries of Health, WHO, UNAIDS, Centers for Disease Control and Prevention, United States Agency for International Development, PEPFAR, non-governmental organisations, HIV experts and members of the community. In addition to the IAPAC database, an Internet search was also done for previously published guidelines using the keywords ’country name’ and ’HIV treatment or antiretroviral’ and ’guidelines’. As PDF versions of national guidelines before 2005 were often not available or links to these guidelines had expired, we restricted the analysis to calculating the time lag in adoption of the WHO 2009, 2013 and 2015 guidelines.

Of the 48 countries in the sub-Saharan African region, we found 92 published national guidelines from 37 countries, representing 98% of regional HIV burden in 2015 (referred to as regional burden henceforth).^2^ For four countries, guidelines recommending ART according to the WHO 2009, 2013 or 2015 guidelines were not available (an old version recommending ART at CD4 count ≤ 200 cells/mm^3^ was available for each country) and they were excluded from further analysis. We screened the remaining 88 guidelines from 33 countries (97% regional burden) and excluded the 29 guidelines that did not contribute to calculating the time to adoption of the WHO 2009, 2013 or 2015 guidelines (they recommended ART at CD4 count ≤ 200 cells/mm^3^ or 250 cells/mm^3^). From the remaining 59 guidelines for 33 countries (97% regional burden), we abstracted information on (1) month and year of publication of the guidelines, and (2) ART eligibility criteria for asymptomatic adults living with HIV. In cases where the month of publication was not known, we assumed that the guidelines were published in June of the same calendar year.

The date of adoption of a WHO guideline was defined as the date of publication of the first national guideline that was consistent with the WHO guideline recommendation. Using the WHO guidelines publication date (i.e. October 2009, June 2013 and September 2015), the time lag was calculated as the number of months taken by a country to adopt the WHO guidelines. The average time lag in WHO guidelines adoption was defined as the total number of months taken to adopt WHO guidelines divided by the number of countries. Delay was calculated separately for the WHO 2009, WHO 2013 and WHO 2015 guidelines according to the available published national guidelines for that period. We also estimated the delay in moving to the WHO 2013 guidelines for all 33 countries by assuming that the countries that are yet to move to CD4 count ≤ 500 cells/mm^3^ will do so by August 2016.

## Results

**FIGURE 1:**
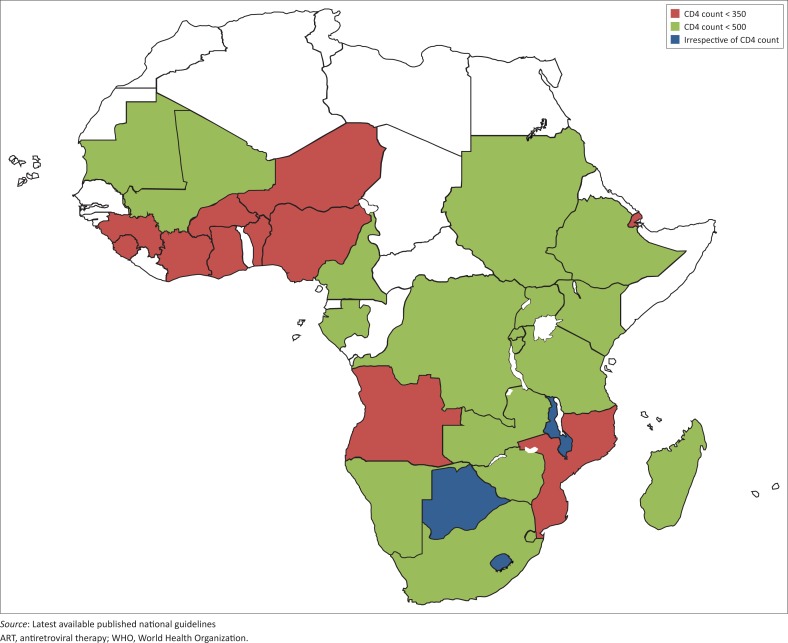
ART eligibility criteria in 33 countries in sub-Saharan Africa as of August 2016 (WHO recommendation: Irrespective of CD4 count). Countries in white are the countries from North Africa and the 15 countries from sub-Saharan Africa without publicly available national guidelines.

According to the latest available published national guidelines from 33 countries, 3 countries (6% regional burden) are recommending ART according to the WHO 2015 guidelines (treatment for all). A total of 19 countries (65% regional burden) are recommending ART according to the WHO 2013 guidelines (CD4 count ≤ 500 cells/mm^3^) and 11 countries (26% regional burden) are still recommending ART according to the WHO 2009 guidelines (CD4 count ≤ 350 cells/mm^3^) (Figure 1).

### Time lag in adopting the WHO 2009 guidelines

**FIGURE 2:**
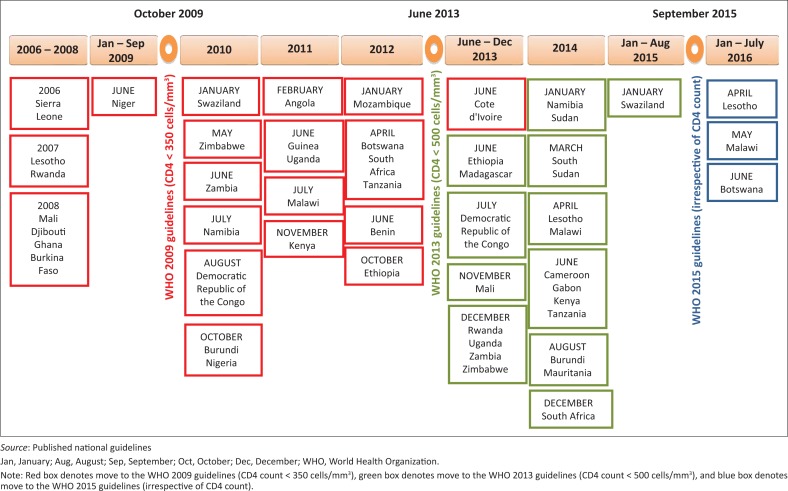
Timeline showing the date of release of the WHO guidelines and national guidelines from 33 countries in sub-Saharan Africa (January 2006–August 2016).

The WHO 2006 guidelines recommended ART at CD4 count ≤ 200 cells/mm^3^, but additionally **considered** treatment at CD4 counts between 200 and 350 cells/mm^3^. Guidelines from eight countries (4% regional burden) released between January 2006 and October 2009 were already recommending ART at CD4 count ≤ 350 cells/mm^3^ before the WHO 2009 guidelines were released. These countries were not included in the analysis of the delay in adopting the WHO 2009 guidelines (Figure 2).

Of the 33 countries, 21 (91% regional burden) are known to have moved to CD4 count ≤ 350 cells/mm^3^ after publication of the WHO 2009 guidelines (Figure 2). On average, these countries took 24 (range 3–56) months to revise their national guidelines in line with the WHO 2009 guidelines (Table 1). For the remaining 4 countries (1% regional burden), national guidelines that adopted the WHO 2009 guidelines were not available.

**TABLE 1:** Time lag in adopting the WHO guidelines published in 2009, 2013 and 2015 in 33 countries in sub-Saharan Africa.

Country	People living with HIV (2015)	Number of people on ART in 2015 (ART coverage)	Estimated AIDS-related deaths (2015)	Estimated new HIV infections (2015)	Date of WHO 2009 guidelines adoption	Time lag (months)	Date of WHO 2013 guidelines adoption	Time lag (months)	Date of WHO 2015 guidelines adoption	Time lag (months)
Angola	320 000	90 204 (29%)	12 000	26 000	Feb 2011	16	Not yet moved to < 500	-	Not yet moved	-
Benin	69 000	33 602 (49%)	2800	4200	Jun 2012	32	Not yet moved to < 500	-	Not yet moved	-
Botswana	350 000	272 972 (78%)	3200	9700	Apr 2012	30	Jun 2016	36	Jun 2016	9
Burkina Faso	95 000	52 304 (55%)	3600	4200	Nov 2008	-	Not yet moved to < 500	-	Not yet moved	-
Burundi	77 000	42 169 (54%)	3000	1200	Oct 2010	12	Aug 2014	14	Not yet moved	-
Cameroon	620 000	168 249 (27%)	33 000	44 000	Jun 2014	56	Jun 2014	12	Not yet moved	-
Côte d’Ivoire	460 000	161 173 (35%)	25 000	25 000	Jun 2013	44	Not yet moved to < 500	-	Not yet moved	-
Democratic Republic of the Congo	370 000	122 268 (33%)	22 000	15 000	Aug 2010	10	Jul 2013	1	Not yet moved	-
Djibouti	9400	1945 (21%)	600	620	Jun 2008	-	Not yet moved to < 500	-	Not yet moved	-
Ethiopia	NA	386 123 (NA)	NA	-	Oct 2012	36	Jun 2013	0	Not yet moved	-
Gabon	47 000	27 037 (58%)	1300	1500	Jun 2014	56	Jun 2014	12	Not yet moved	-
Ghana	270 000	94 047 (34%)	13 000	13 000	Jun 2008	-	Not yet moved to < 500	-	Not yet moved	-
Guinea	120 000	33 525 (29%)	4600	7600	Jun 2011	20	Not yet moved to < 500	-	Not yet moved	-
Kenya	1 500 000	897 644 (59%)	36 000	78 000	Nov 2011	25	Jun 2014	12	Not yet moved	-
Lesotho	310 000	129 127 (42%)	9 900	18 000	Jun 2007	-	Apr 2014	10	Apr 2016	7
Madagascar	48 000	1234 (3%)	3200	6300	Unknown	-	Jun 2013	0	Not yet moved	-
Malawi	980 000	595 186 (61%)	27 000	33 000	Jul 2011	21	Apr 2014	10	May 2016	8
Mali	120 000	34 974 (28%)	6500	10 000	Mar 2008	-	Nov 2013	5	Not yet moved	-
Mauritania	14 000	2458 (18%)	960	730	Unknown	-	Aug-14	14	Not yet moved	-
Mozambique	1 500 000	802 659 (53%)	39 000	81 000	Jan 2012	27	Not yet moved to < 500	-	Not yet moved	-
Namibia	210 000	144 496 (69%)	3100	7800	Jul 2010	9	Jan 2014	7	Not yet moved	-
Niger	49 000	12 887 (26%)	3600	2400	Jun 2009	-	Not yet moved to < 500	-	Not yet moved	-
Nigeria	3 500 000	828 867 (24%)	180 000	250 000	Oct 2010	12	Not yet moved to < 500	-	Not yet moved	-
Rwanda	200 000	158 728 (79%)	2900	7500	Jul 2007	-	Dec 2013	6	Not yet moved	-
Sierra Leone	51 000	14 041 (27%)	2500	2500	Aug 2006	-	Not yet moved to < 500	-	Not yet moved	-
South Africa	7 000 000	3 384 160 (48%)	180 000	380 000	Apr 2012	30	Dec 2014	18	Not yet moved	-
South Sudan	180 000	19 553 (11%)	12 000	15 000	Unknown	-	Mar 2014	9	Not yet moved	-
Sudan	56 000	4388 (8%)	3000	5600	Unknown	-	Jan 2014	7	Not yet moved	-
Swaziland	220 000	147 274 (67%)	3800	11 000	Jan 2010	3	Jan 2015	19	Not yet moved	-
Tanzania	1 400 000	740 078 (53%)	36 000	54 000	Apr 2012	30	Jun 2014	12	Not yet moved	-
Uganda	1 500 000	834 931 (57%)	28 000	83 000	Jun 2011	20	Dec 2013	6	Not yet moved	-
Zambia	1 200 000	758 646 (63%)	20 000	60 000	Jun 2010	8	Dec 2013	6	Not yet moved	-
Zimbabwe	1 400 000	878 461 (62%)	29 000	64 000	May 2010	7	Dec 2013	6	Not yet moved	-
**Average time lag**	-	-	-	-	-	**24**	-	**10**	-	**8**

NA, not applicable; Jan, January; Feb, February; Mar, March; Apr, April; Jun, June; Jul, July; Aug, August; Oct, October; Nov, November; Dec, December.

### Time lag in adopting the WHO 2013 guidelines

None of the countries in sub-Saharan Africa moved to the CD4 ≤ 500 cells/mm^3^ recommendation before the release of the WHO 2013 guidelines (Figure 2). Of the 33 countries, 22 (92% regional burden) moved to CD4 count ≤ 500 cells/mm^3^ after publication of the WHO 2013 guidelines (or earlier) (Figure 2). These countries took an average of 10 (range 0–36) months to revise their national guidelines in line with the WHO 2013 recommendations (Table 1). Eleven countries (26% regional burden) have not yet adopted the WHO 2013 guidelines. Assuming these 11 countries adopt the WHO 2013 guidelines by August 2016, average time lag would be 19 (range 0–38) months.

### Time lag in adopting the WHO 2015 guidelines

None of the countries in sub-Saharan Africa moved to ART irrespective of CD4 count before the release of the WHO 2015 guidelines (Figure 2). Of the 33 countries, 3 (6% regional burden) moved to ART irrespective of CD4 count after publication of the WHO 2015 guidelines (Figure 2). These countries took an average of 8 (range 7–9) months to revise their national guidelines in line with the WHO 2015 recommendations.

## Discussion

The national treatment guidelines are vital tools for programme design and management of people living with HIV. Timely adoption of international best practices and normative guidelines as national policies, and successful dissemination and implementation of these policies, are critical for accelerated access to ART. Guideline revisions can be complex and often involve consensus building around technical and operational issues and negotiations with internal and external stakeholders regarding budget prioritisation. Our study suggests that adoption of national treatment guidelines from sub-Saharan African countries has been very slow, with countries taking an average of 2 years to move to CD4 ≤ 350 cells/mm^3^ after release of the WHO 2009 guidelines. It has been 3 years since the release of the WHO 2013 guidelines, but 11 countries, including many high-burden countries such as Nigeria and Mozambique, have not yet adopted these guidelines.

As of August 2016, after 11 months, only three countries in the region had adopted and published national guidelines reflecting the WHO 2015 recommendations, which were published in September 2015. Recent public announcements about plans to adopt ’test and treat’ suggest that adoption of the WHO 2015 guidelines may be more rapid than for the WHO 2009 and 2013 guidelines.^21^ However, implementation of national guidelines takes additional time which threatens to increase the delay to many years before the WHO 2015 guidelines become actual practice in the countries. Understanding these delays and considering ways to shorten the time lag from science to guideline adoption to delivery of HIV services is essential to more rapidly expand access to treatment for people living with HIV and achieving the 90-90-90 targets.

Countries with low HIV prevalence and higher HIV spending are more likely to adopt the WHO guidelines.^22^ This is evident from the global trends – treatment for all has been the standard of care in 18 high- and middle-income countries with low HIV prevalence for some years.^20^ On the other hand, Lesotho, Malawi and Botswana have adopted this recommendation recently.^20^ Also, patterns of HIV spending in high-burden countries do not consistently reflect the new science around ‘treatment as prevention’. Many sub-Saharan African countries are allocating less than 50% of their total HIV spending on care and treatment.^23^ HIV spending on ART per person living with HIV is below US$200 in most of these countries.^23^

Adoption and implementation of the WHO 2015 guidelines will pay off with improvement in ART coverage and reductions in illness, AIDS-related deaths and HIV transmission, and save health and other costs.^5,6,7^ With nearly 2200 AIDS-related deaths and 3800 new infections each day,^2^ translating the new science into action more quickly needs to become a major public health priority for the sub-Saharan African region. The countries in this region, which are already struggling to provide ART, will require additional funds and/or more efficient resource utilisation to make a timely move to ‘treatment for all’. Given that international funding has flat-lined, countries will need to commit more funds from domestic or innovative financing sources and allocate more resources for care and treatment.^21^ Additionally, investments in innovative service delivery models (e.g. community-based testing and drug dispensing, nurse-initiated ART), decreased use of CD4 count for ART initiation and monitoring, and advances in antiretroviral regimens and technology will allow more people to be on treatment with the same or even less resources.

### Limitations of the study

Our study has several limitations. We concentrated solely on published national guidelines to determine the date of adoption of the WHO recommendations. Written policies might not reflect practice in countries or might have been published after a recommendation was implemented, suggesting that we might have overestimated the time lag in some cases. Even though we did a comprehensive search for all national guidelines, we might have missed some guidelines; and some guidelines may be outdated or in the process of being updated.

## Conclusion

Since 2000, HIV treatment has dramatically changed the natural history of the HIV epidemic. The new science showing the benefits of treatment for all^5,6,7^ along with other prevention interventions has provided the opportunity to end AIDS by 2030. However, our study shows that it takes years for national policies to reflect the latest science and international standard of care. Even if published guidelines reflect new normative standards, implementation and access to the best care may lag. AIDS response is at a crucial juncture; the new science around treatment and the health and human rights obligation of governments calls for strategic use of resources to rapidly translate new science regarding HIV treatment into action.^24,25^ An estimated 13.4m people living with HIV are eligible but not on treatment.^2^ The HIV epidemic is by no means over, and there is an urgent need to move away from the traditional ’one step at a time, business as usual’ approach to guidelines production, dissemination and revision. The present analysis, which benchmarks the current pace of policy adoption, may help global and national accountability to expedite the adoption and implementation of ‘treatment for all’ in sub-Saharan Africa to win the fight against HIV.
